# The Cinchona Tree

**Published:** 1867-06

**Authors:** 


### THE CINCHONA TREE.

On motion of Dr. Atkinson, the President was directed to appoint a committee to memorialize Congress, relative to the cultivation of the cinchona tree in this country.

